# Utility of Quantitative Sensory Testing and Screening Tools in Identifying HIV-Associated Peripheral Neuropathy in Western Kenya: Pilot Testing

**DOI:** 10.1371/journal.pone.0014256

**Published:** 2010-12-08

**Authors:** Deanna Cettomai, Judith Kwasa, Caroline Kendi, Gretchen L. Birbeck, Richard W. Price, Elizabeth A. Bukusi, Craig R. Cohen, Ana-Claire Meyer

**Affiliations:** 1 Center for Microbiology Research, Kenya Medical Research Institute, Nairobi, Kenya; 2 Department of Neurology, University of California San Francisco, San Francisco, California, United States of America; 3 International Neurologic and Psychiatric Epidemiology Program, Michigan State University, East Lansing, Michigan, United States of America; 4 Department of Obstetrics, Gynecology, and Reproductive Sciences, University of California San Francisco, San Francisco, California, United States of America; Tulane University, United States of America

## Abstract

**Background/Aim:**

Neuropathy is the most common neurologic complication of HIV but is widely under-diagnosed in resource-constrained settings. We aimed to identify tools that accurately distinguish individuals with moderate/severe peripheral neuropathy and can be administered by non-physician healthcare workers (HCW) in resource-constrained settings.

**Methods:**

We enrolled a convenience sample of 30 HIV-infected outpatients from a Kenyan HIV-care clinic. A HCW administered the Neuropathy Severity Score (NSS), Single Question Neuropathy Screen (Single-QNS), Subjective Peripheral Neuropathy Screen (Subjective-PNS), and Brief Peripheral Neuropathy Screen (Brief-PNS). Monofilament, graduated tuning fork, and two-point discrimination examinations were performed. Tools were validated against a neurologist's clinical assessment of moderate/severe neuropathy.

**Results:**

The sample was 57% male, mean age 38.6 years, and mean CD4 count 324 cells/µL. Neurologist's assessment identified 20% (6/30) with moderate/severe neuropathy. Diagnostic utilities for moderate/severe neuropathy were: Single-QNS - 83% sensitivity, 71% specificity; Subjective-PNS-total - 83% sensitivity, 83% specificity; Subjective-PNS-max and NSS - 67% sensitivity, 92% specificity; Brief-PNS - 0% sensitivity, 92% specificity; monofilament - 100% sensitivity, 88% specificity; graduated tuning fork - 83% sensitivity, 88% specificity; two-point discrimination - 75% sensitivity, 58% specificity.

**Conclusions:**

Pilot testing suggests Single-QNS, Subjective-PNS, and monofilament examination accurately identify HIV-infected patients with moderate/severe neuropathy and may be useful diagnostic tools in resource-constrained settings.

## Introduction

Peripheral neuropathy is the most common neurologic complication of HIV but is widely under-recognized and under-treated in resource-constrained settings.[1] Task-shifting, delegating healthcare tasks to less specialized healthcare workers, is common in many such locations as a result of the scale-up of antiretroviral programs.[2] Simple inexpensive diagnostic tools that can be administered by non-physician healthcare workers may improve recognition of neuropathy in resource-constrained settings.

Several screening tools and quantitative sensory testing (QST) methods, including the monofilament, Rydel-Seiffer graduated tuning fork, and two-point discriminator, have been shown to accurately identify individuals with neuropathy.[3]–[8] However, these tools have been almost exclusively validated in high-income countries by specialized physicians. Furthermore, none includes a functional status assessment which may be important to identify individuals with a moderate to severe neuropathy in need of intervention. Therefore, we developed the Neuropathy Severity Score (NSS), a novel diagnostic tool with a functional status assessment. We then evaluated the utility of the NSS, QST, and other previously validated diagnostic tools in identifying patients with moderate to severe peripheral neuropathy in a resource-constrained setting.

## Methods

The Kenya Medical Research Institute National Ethical Review Committee and University of California San Francisco Committee on Human Research approved this study. Written informed consent was obtained from a convenience sample of 30 HIV-infected outpatients over 18 years of age between September and October 2009 at Family AIDS Care and Education Services, an HIV-care clinic in Kisumu, Kenya.

Nurses and clinical officers administered the diagnostic tool under investigation (Appendix S1) to each participant. No training was provided in its administration. The tool's components were derived from the Brief Peripheral Neuropathy Screen (Brief-PNS) [3], Subjective Peripheral Neuropathy Screen (Subjective-PNS) [4], Single Question Neuropathy Screen (Single-QNS) [5] and the physical function scale from the Medical Outcomes Study Core Survey Instrument, RAND Health (www.rand.org/health/surveys_tool/mos/). Scores for Brief-PNS, Subjective-PNS, Single-QNS, and NSS (derived from Subjective-PNS and physical function scale) were obtained (Appendix S2). The nurses and clinical officers administering the tool were blinded to the neurologist's assessment. Study staff performed QST examination with monofilament[9], [10], graduated tuning fork [7], [11]–[13], and two-point discriminator [8] on each participant (Appendix S3). The order of QST examinations was decided using a random number table. Study staff administering QST were blinded to the neurologist's clinical assessment in all but three cases where staffing constraints prevented blinded administration. Eighteen different nurses and clinical officers were used in the administration of the diagnostic tool; two different study staff administered QST. Intra- and inter-observer variability for QST examinations were not investigated in this study but have been previously reported.[13]–[15]

A neurologist performed a standardized clinical assessment based on AIDS Clinical Trials Group (ACTG) protocol.[16] The neurologist was blinded to the results of the diagnostic tool, and, in all but three cases mentioned above, blinded to the QST results. Peripheral neuropathy was defined as the presence of ≥1 sign of neuropathy - reduced sensation to pinprick, vibration, or reduced ankle reflexes—definitions drawn from the ACTG protocol.[16] For analysis, the study sample was dichotomized into moderate/severe and mild/no peripheral neuropathy. Moderate peripheral neuropathy was defined as pinprick diminished to the ankles or vibration reduced to <5 seconds at the great toe.

Demographic variables included mean household wealth, calculated from self-report of household possessions, and food insecurity, defined as eating ≤1 meal on any day in the week prior to enrollment.

Statistical analyses were performed using Stata 10.0 (StataCorp, College Station, Texas). Demographic and clinical characteristics were compared using Fisher's exact tests and t-tests. Diagnostic utility of the screening tools and QST was determined using the neurologist's clinical assessment of moderate/severe neuropathy as the gold standard. Sensitivity, specificity, predictive values, likelihood ratios, and accuracy were calculated. Receiver operating characteristic (ROC) curves were generated for NSS, Subjective-PNS, and two-point discrimination examinations. Kappa statistic measured agreement on tool components assessed by the neurologist and non-physician healthcare workers.

## Results

The mean age of participants was 38.6 (±10.5 SD) years, and 17 (57%) were male. A majority of patients were WHO Stage 3 or 4, had CD4 counts <350 cells/µL, and were currently taking antiretroviral medications. Based on the neurologist's gold standard clinical examination, 16 (53%) of participants had neuropathy with 6 (20%) individuals having moderate or severe neuropathy (Table 1). All participants with moderate/severe neuropathy had previously used stavudine (d4T), and 2 (33%) were taking d4T at study enrollment. Compared to those with mild/no neuropathy, participants with moderate/severe neuropathy had significantly lower mean household wealth and were significantly more likely to have discontinued d4T due to neuropathy.

**Table 1:** Comparison of demographic and clinical characteristics between participants with mild or no peripheral neuropathy and those with moderate or severe peripheral neuropathy*.

	None or Mild (n = 24)	Moderate or Severe (n = 6)	p†
Age	37.5 (11.2)	43 (5.8)	0.26
Male	54% (13)	67% (4)	0.67
Household wealth (USD) ‡	888 (1384)	217 (97)	0.03
Food insecurity**	25% (6)	0% (0)	0.30
BMI	21.3 (2.8)	22.3 (3.6)	0.47
CD4 nadir	218 (158)	114 (74)	0.12
Current CD4	316 (229)	353 (126)	0.7
WHO Stage 3 or 4	54% (13)	83% (5)	0.36
Time since HIV diagnosis (months)	10.4 (0.88)	10.3 (0.52)	0.91
Ever used d4T	67% (16)	100% (6)	0.16
Current d4T use	46% (11)	33% (2)	0.67
Discontinued d4T due to peripheral neuropathy	8% (2)	67% (4)	0.007
Ever used isoniazid	42% (10)	50% (3)	1.0
Ever used ddI	4% (1)	0% (0)	1.0
Any alcohol use	17% (4)	0% (0)	0.56
Abnormal thyroid exam	0% (0)	17% (1)	0.20
Mean corpuscular volume ever >100 fL	29% (7)	50% (3)	0.37
RPR ever positive	8% (2)	0% (0)	1.0
Random blood glucose ever >200 mg/dL	0% (0)	0% (0)	----
Fasting blood glucose ever >120 mg/dL	0% (0)	0% (0)	----
Creatinine ever >240 µmol/L (2.7 mg/dL)	0% (0)	0% (0)	----
ALT >80 U/L	0% (0)	0% (0)	----

Abbreviations: USD: United States Dollars; BMI: Body Mass Index; WHO: World Health Organization; d4T: stavudine; ddI: didanosine; RPR: rapid plasma regain; ALT: alanine transaminase.

*All variables presented as [mean (SD)] or [% (n)],

† p-values calculated from two-sample t-tests of means and of Fisher's exact tests of proportions.

‡ Household wealth calculated from patient self-report of household possessions.

**Food insecurity defined as eating only 1 meal per day or having gone ≥1 day without eating in the past one week.

Diagnostic utility of the screening tools and QST in identifying participants with moderate/severe neuropathy is presented in Table 2. NSS and Subjective-PNS-max score performed identically and were the most specific, while Subjective-PNS-total had the highest area under the ROC curve. Single-QNS was 83% sensitive and 71% specific. Brief-PNS was 0% sensitive and 92% specific. Agreement between ankle reflexes performed by non-physician healthcare workers and by a neurologist was poor (kappa = 0.04; 95% CI [−0.19, 0.37]; p = 0.40). Monofilament examination was 100% sensitive, 88% specific, and correctly classified 90% of participants (Table 2). Tuning fork examination was 83% sensitive and 88% specific while two-point discrimination had a sensitivity of 75% and specificity of 58%.

**Table 2:** Diagnostic utility of peripheral neuropathy screening tools and quantitative sensory testing in detecting moderate to severe peripheral neuropathy.

	Sensitivity [95%CI]	Specificity [95%CI]	PPV	NPV	Acc	LR+	LR-	AUC	Cutoff
Neuropathy Severity Score	66.7 [29, 104]	91.7 [81, 103]	66.7	91.7	86.7	8	0.4	0.83	≥6
Subjective-PNS – Maximum Score	66.7 [29, 104]	91.7 [81, 103]	66.7	91.7	86.7	8	0.4	0.83	≥5
Subjective-PNS) - Total Score	83.3 [53, 113]	83.3 [68, 98]	55.6	95.2	83.3	5	0.2	0.86	≥6
Single Question Neuropathy Screen	83.3 [53, 113]	70.8 [53, 89]	41.7	94.4	73.3	2.9	0.2	0.77	----
Brief-PNS	0 [0,0]	91.7 [81, 103]	0	78.6	73.3	0	1.1	0.46	----
Monofilament	100 [100,100]	87.5 [74, 101]	66.7	100	90	8	0	0.94	≥2
Graduated Tuning Fork	83.3 [53, 113]	87.5 [74, 101]	62.5	95.4	86.7	6.7	0.2	0.85	----
Two-Point Discrimination	75 [40, 109]	58.3 [39, 78]	23.1	93.3	60.7	1.8	0.4	0.70	≥4

Abbreviations: Acc: Accuracy; PNS: Peripheral Neuropathy Screen.

## Discussion

The overall prevalence of neuropathy in our sample was comparable to previously published results from similar settings.[5] While many previously established risk factors did not differ significantly between participants with moderate/severe neuropathy and those with mild/no neuropathy, mean household wealth did. Mean household wealth may be a proxy measure for another factor, such as nutritional status or the opportunity cost of accessing medical care, including missed wages or transport costs. Further investigation in a larger sample is warranted.

In our sample, NSS, Single-QNS, Subjective-PNS-total, and Subjective-PNS-max performed well and had adequate sensitivity and specificity to be useful as diagnostic tools in settings with high neuropathy prevalence. Even Single-QNS, the simplest tool, had a sensitivity over 80% and a specificity greater than 70%. A Zambian study conducted by a different research group using different methodology found Single-QNS was 96% sensitive and 80% specific.[5] These results provide support for routine use of Single-QNS in resource-constrained settings.

NSS, which was developed to incorporate functional status assessment into a diagnostic tool, did not improve gradation of neuropathy severity as we anticipated. This may be due to small sample size or insensitivity of the tool. Brief-PNS performed quite poorly in our sample, (sensitivity = 0%), which is likely the result of poor agreement of ankle reflexes between non-physician healthcare workers and the gold standard examination. Of note, we did not provide training in evaluating ankle reflexes because our goal was to identify a tool which would be feasible to scale up across sub-Saharan Africa where such training would not often be available.

Monofilament and graduated tuning fork also performed very well and may be useful in research and selected clinical settings in resource-constrained locations. QST offer objective measures of neuropathy that are less expensive, necessitate less specialized equipment, and require less training as compared to other techniques such as nerve conduction studies. In addition, some methods have been shown to predict health outcomes; for example, monofilament examination has shown significant predictive power in identifying individuals with neuropathy due to diabetes and leprosy who are at greatest risk of experiencing foot ulcerations.[6], [17] However, these techniques must be validated in resource-constrained settings to ensure feasibility and accuracy.

This pilot study has several limitations. Prevalence estimates for HIV-associated neuropathy should be interpreted with caution due to small sample size, use of convenience sampling, and inclusion of only outpatients enrolled in routine care. Additionally, neuropathies identified in this population are likely due to both HIV and other etiologies, as we were unable to definitively rule out other causes. Finally, due to resource limitations, we were unable to include other objective measures of neuropathy in our gold standard, such as nerve conduction studies, computerized QST, or intra-epidermal nerve fiber densities. Nerve conduction studies can only be performed in Nairobi, Kenya, six to eight hours drive from our study site. Computerized QST technology and intra-epidermal nerve fiber densities are not currently available in Kenya. Nevertheless, a neurologist's clinical assessment has been used widely in other studies and is an accepted gold standard [18].

A major strength of our study is its applicability to everyday clinical practice in resource-constrained settings. Excluding QST, we did not provide specialized equipment or training to non-physician healthcare workers who administered the diagnostic tools. As such, our results are likely replicable in similar resource-constrained settings, so comparable results would be expected with widespread implementation of these tools. However, these results are preliminary and require further validation among a larger sample before generalizing more broadly to other patient populations.

## Supporting Information

Appendix S1 Neuropathy Diagnostic Tool.
(0.05 MB DOC)

Appendix S2 Scoring of individual screening tools and quantitative sensory tests.
(0.08 MB DOC)

Appendix S3 Quantitative Sensory Testing Protocol and Normative Values.
(0.06 MB DOC)
