# Supplementary material for: Utility of Quantitative Sensory Testing and Screening Tools in Identifying HIV-Associated Peripheral Neuropathy in Western Kenya: Pilot Testing
Source: PLoS One. 2010 Dec 8;5(12):e14256. doi: 10.1371/journal.pone.0014256 (PMC2999535; doi:10.1371/journal.pone.0014256)
Supplement: Appendix S1 — Neuropathy Diagnostic Tool. (0.05 MB DOC) [file pone.0014256.s001.doc]

Name of person administering tool: __________________________________

| **What is your position at FACES?** |  |
| --- | --- |
| - Community Health Worker | - Clinical Officer |
| - Clinic Community Health Assistant | - Medical Officer |
| - Nurse | - Other, please specify ____________________ |

| **HIV NEUROPATHY DIAGNOSTIC TOOL** | | | | | | | |
| --- | --- | --- | --- | --- | --- | --- | --- |
|  | | | | | | | |
| **PART A** | | | | | | | |
| Please ask the patient to rate the **current** severity of each of the following symptoms on a scale of 1 (mild) to 10 (most severe). Use 0 if the symptom is not present. | | | | | | | |
|  | | | **Right**  **(0 – 10)** | | **Left**  **(0-10)** | | |
| Pain, aching or burning in the feet or legs | | |  | |  | | |
| “Pins and needles” in feet or legs | | |  | |  | | |
| Numbness (lack of feeling in the feet or legs) | | |  | |  | | |
| **IF all of the above are 0, skip to Part C** | | | | | | | |
|  | | | | | | | |
|  | **PART B** | | | | | |  |
|  | Does the numbness, pain, burning or tingling in your legs limit you in these activities? If so, how much? (Tick the appropriate box.) | | | | | |  |
|  |  | **Yes, limited**  **a lot** | | **Yes, limited**  **a little** | | **No, not limited at all** |  |
|  | Vigorous activities, such as lifting heavy objects, participating in football, farming, washing clothes by hand, carrying water |  | |  | |  |  |
|  | Moderate activities, such as moving a table, light household chores, riding a bicycle, walking |  | |  | |  |  |
|  | Climbing one flight of stairs or a small hill |  | |  | |  |  |
|  | Walking 50 meters (1/2 a football pitch) |  | |  | |  |  |
|  | | | | | | | |
| **PART C** | | | | | | | |
| Please check the patient’s ankle reflexes (i.e. ankle jerks).  0 = absent 1 = decreased 2 = normal 3 = increased 4 = clonus | | | | | | | |
|  | | | **Right**  **(0 – 4)** | | **Left**  **(0 – 4)** | | |
| Ankle reflexes | | |  | |  | | |
