# Supplementary material for: Utility of Quantitative Sensory Testing and Screening Tools in Identifying HIV-Associated Peripheral Neuropathy in Western Kenya: Pilot Testing
Source: PLoS One. 2010 Dec 8;5(12):e14256. doi: 10.1371/journal.pone.0014256 (PMC2999535; doi:10.1371/journal.pone.0014256)
Supplement: Appendix S2 — Scoring of individual screening tools and quantitative sensory tests. (0.08 MB DOC) [file pone.0014256.s002.doc]

| **Portion of Diagnostic Tool Utilized** | **Calculation of Score** |
| --- | --- |
| ***Neuropathy Severity Score (NSS)*** | |
| *Part A: Subjective Symptoms* | Highest score on any single symptom item (0-10) |
| *Part B: Functional Status* | Sum of functional status items (0-8) |
| ***Total Score*** | ***0-18*** |
| ***Subjective Peripheral Neuropathy Screen (Subjective-PNS) – Maximum Score [1]*** | |
| *Part A: Subjective Symptoms* | Highest score on any single symptom item |
| ***Total Score*** | ***0-10*** |
| ***Subjective Peripheral Neuropathy Screen (Subjective-PNS) - Total Score [1]*** | |
| *Part A: Subjective Symptoms* | Sum of scores for each symptom item |
| ***Total Score*** | ***0-60*** |
| ***Single Question Neuropathy Screen (Single-QNS) [2]*** | |
| *Part A: Subjective Symptoms* | Dichotomous: Abnormal if reports any symptoms (i.e. > 1 on any item) |
| ***Total Score*** | ***Dichotomous*** |
| ***Brief Peripheral Neuropathy Screen (Brief-PNS) [3]*** | |
| *Part A: Subjective Symptoms* | Dichotomous: Abnormal if reports any symptoms (i.e. > 1 on any item) |
| *Part C: Ankle Reflexes* | Dichotomous; Abnormal if ankle reflexes are diminished (i.e. < 2+) |
| ***Total Score*** | ***Dichotomous:*** *Abnormal if both of above criteria are met* |
| ***Monofilament (MF) [4,5]*** | |
| *Administration Protocol* | 10-g MF applied in triplicate to plantar surface of hallux, 1st MT, 3rd MT and 5th MT and patient asked to state if it was felt on left or right foot. |
| ***Scoring*** | ***Abnormal if > 1 trial is not perceived at a given site*** |
| ***Rydel-Seiffer Graduated Tuning Fork (TF) [6-9]*** | |
| *Administration Protocol* | TF applied to distal interphalangeal joint of hallux in triplicate bilaterally. Patient stated when vibration ceased, and threshold was value at intersection of virtual triangle at that time. |
| ***Scoring*** | ***Abnormal if threshold was less than normative values on > 1 trial*** |
| ***Two-Point Discrimination (2PD) [10]*** | |
| *Administration Protocol* | Plantar surface of hallux, 1st MT, and heel were tested in triplicated by applying consecutively spaced rods. Patient stated whether they perceived 1 or 2 rods, and threshold was smallest distance for which 2 points were felt. |
| **Scoring** | ***Abnormal if threshold was greater than normative value on > 1 trial*** |

References:

1. Venkataramana AB, Skolasky RL, Creighton JA, McArthur JC (2005) Diagnostic utility of the subjective peripheral neuropathy screen in HIV-infected persons with peripheral sensory polyneuropathy. AIDS Read 15: 341-344, 348-349, 354.

2. Kandiah P, Atadzhanov M, Kvalsund M, Birbeck G Evaluating the diagnostic capacity of a single-question neuropathy screen (SQNS) in HIV positive Zambian adults. J Neurol Neurosurg Psychiatry In press.

3. Cherry C, Wesselingh S, Lal L, McArthur J (2005) Evaluation of a clinical screening tool for HIV-associated sensory neuropathies. Neurology 65: 1778-1781.

4. Diamond J, Mueller M, Delitto A, Sinacore D (1989) Reliability of a diabetic foot evaluation. Phys Ther 69: 797-802.

5. Arseculeratne YM, Cherry GW (2003) Sensory testing in patients with chronic venous leg ulcers using a 10 g Owen Mumford monofilament. J Wound Care 12: 215-217.

6. Martina IS, van Koningsveld R, Schmitz PI, van der Meche FG, van Doorn PA (1998) Measuring vibration threshold with a graduated tuning fork in normal aging and in patients with polyneuropathy. European Inflammatory Neuropathy Cause and Treatment (INCAT) group. J Neurol Neurosurg Psychiatry 65: 743-747.

7. Thivolet C, el Farkh J, Petiot A, Simonet C, Tourniaire J (1990) Measuring vibration sensations with graduated tuning fork. Simple and reliable means to detect diabetic patients at risk of neuropathic foot ulceration. Diabetes Care 13: 1077-1080.

8. Merkies IS, Schmitz PI, van der Meche FG, van Doorn PA (2000) Reliability and responsiveness of a graduated tuning fork in immune mediated polyneuropathies. The Inflammatory Neuropathy Cause and Treatment (INCAT) Group. J Neurol Neurosurg Psychiatry 68: 669-671.

9. Kastenbauer T, Sauseng S, Brath H, Abrahamian H, Irsigler K (2004) The value of the Rydel-Seiffer tuning fork as a predictor of diabetic polyneuropathy compared with a neurothesiometer. Diabet Med 21: 563-567.

10. Periyasamy R, Manivannan M, Narayanamurthy VB (2008) Changes in Two Point Discrimination and the law of mobility in Diabetes Mellitus patients. J Brachial Plex Peripher Nerve Inj 3: 3.
