# Supplementary material for: Utility of Quantitative Sensory Testing and Screening Tools in Identifying HIV-Associated Peripheral Neuropathy in Western Kenya: Pilot Testing
Source: PLoS One. 2010 Dec 8;5(12):e14256. doi: 10.1371/journal.pone.0014256 (PMC2999535; doi:10.1371/journal.pone.0014256)
Supplement: Appendix S3 — Quantitative Sensory Testing Protocol and Normative Values. (0.06 MB DOC) [file pone.0014256.s003.doc]

Monofilament examination (Bailey Instruments LTD, Manchester UK) was performed in triplicate using a 10-g monofilament on the plantar surface of the hallux, 1^st^ metatarsal head, 3^rd^ metatarsal head, and 5^th^ metatarsal head of each foot. The order of sites was determined using a random number table. Participants were asked to state ‘left’ or ‘right’ upon detecting the monofilament. A correct response was recorded if the correct side was stated immediately after application of the monofilament. The examination was abnormal if the monofilament was not detected on >1 trial at >2 sites.[1-3]

Vibration examination was performed using the Rydel-Seiffer graduated tuning fork (US Neurologicals; www.usneurologicals.com). This is a 64Hz tuning fork which bears calibrated weights imprinted with a triangle and an arbitrary scale ranging from 0 (minimum score) to 8 (maximum score) at the end of each arm. When the arms are swinging, the triangles on the weights appear double, and the intersection of these virtual triangles moves in an exponential manner from 0 to 8 as the vibration amplitude of the arms decreases.[4,5] The instrument was applied perpendicularly resting on its own weight to the distal interphalangeal joint of the hallux and the participant was asked to indicate when the vibration was no longer detectable. The vibration threshold was recorded as the nearest value (0.5 unit increments) to the intersection of the virtual triangles when vibration perception ceased. The test was repeated in triplicate and was abnormal if the value at either toe was less than previously published normative values (minimum values for normal exam, stratified by age: < 40 years – 4.5; 41-60 years – 4.0; 61-85 years – 3.5; > 85 years – 3.0).[4,6]

Two-point discrimination was performed using a Disk-Criminator (MacKinnon^TM^), a set of two discs with a series of pairs of metal rods spaced at varying intervals ranging from 1mm to 25mm. The plantar surface of the hallux, 1^st^ metatarsal head, and heel were each tested in triplicate by applying consecutively spaced metal rods perpendicularly to the skin surface. Participants stated whether they perceived one or two rods. The two-point discrimination threshold was the smallest value at which two rods were perceived. The examination was abnormal if the values at >4 sites were greater than previously established normative values (maximum values for normal exam by exam location: 5 mm at the hallux; 15 mm at the 1^st^ metatarsal head; 20 mm at the heel).[7]

References:

1. Arseculeratne YM, Cherry GW (2003) Sensory testing in patients with chronic venous leg ulcers using a 10 g Owen Mumford monofilament. J Wound Care 12: 215-217.

2. Kastenbauer T, Sauseng S, Brath H, Abrahamian H, Irsigler K (2004) The value of the Rydel-Seiffer tuning fork as a predictor of diabetic polyneuropathy compared with a neurothesiometer. Diabet Med 21: 563-567.

3. Diamond J, Mueller M, Delitto A, Sinacore D (1989) Reliability of a diabetic foot evaluation. Phys Ther 69: 797-802.

4. Martina IS, van Koningsveld R, Schmitz PI, van der Meche FG, van Doorn PA (1998) Measuring vibration threshold with a graduated tuning fork in normal aging and in patients with polyneuropathy. European Inflammatory Neuropathy Cause and Treatment (INCAT) group. J Neurol Neurosurg Psychiatry 65: 743-747.

5. Thivolet C, el Farkh J, Petiot A, Simonet C, Tourniaire J (1990) Measuring vibration sensations with graduated tuning fork. Simple and reliable means to detect diabetic patients at risk of neuropathic foot ulceration. Diabetes Care 13: 1077-1080.

6. Merkies IS, Schmitz PI, van der Meche FG, van Doorn PA (2000) Reliability and responsiveness of a graduated tuning fork in immune mediated polyneuropathies. The Inflammatory Neuropathy Cause and Treatment (INCAT) Group. J Neurol Neurosurg Psychiatry 68: 669-671.

7. Periyasamy R, Manivannan M, Narayanamurthy VB (2008) Changes in Two Point Discrimination and the law of mobility in Diabetes Mellitus patients. J Brachial Plex Peripher Nerve Inj 3: 3.
